# Three Functions of Cadherins in Cell Adhesion

**DOI:** 10.1016/j.cub.2013.06.019

**Published:** 2013-07-22

**Authors:** Jean-Léon Maître, Carl-Philipp Heisenberg

**Affiliations:** 1 EMBL, Meyerhofstraße 1, 69117 Heidelberg, Germany; 2 IST Austria, Am Campus 1, 3400 Klosterneuburg, Austria

## Abstract

Cadherins are transmembrane proteins that mediate cell–cell adhesion in animals. By regulating contact formation and stability, cadherins play a crucial role in tissue morphogenesis and homeostasis. Here, we review the three major functions of cadherins in cell–cell contact formation and stability. Two of those functions lead to a decrease in interfacial tension at the forming cell–cell contact, thereby promoting contact expansion — first, by providing adhesion tension that lowers interfacial tension at the cell–cell contact, and second, by signaling to the actomyosin cytoskeleton in order to reduce cortex tension and thus interfacial tension at the contact. The third function of cadherins in cell–cell contact formation is to stabilize the contact by resisting mechanical forces that pull on the contact.

## Main Text

### Introduction

The ability of cells to adhere to one another is a fundamental property in the evolution of multicellularity. Cadherins are transmembrane cell–cell adhesion molecules conserved among metazoan organisms, which play essential roles in tissue morphogenesis and homeostasis 1, 2, 3, 4, 5. During morphogenesis, tissues can change in size and shape, and form distinct cell layers. Cadherins function in tissue morphogenesis by controlling both cell–cell adhesion and cell signaling. For example, cadherins are involved in determining cell shape and position within the ommatidium of the *Drosophila melanogaster* retina [1]. Cadherins have also been implicated in germ cell positioning and migration in zebrafish and *Caenorhabditis elegans* 4, 6, oocyte positioning within the egg-chamber of *Drosophila* [7], epithelial folding 3, 8, and mesoderm/endoderm cell internalization in *Drosophila* and *Caenorhabditis elegans* 2, 9. Cells also use cadherins to mediate signals that can control cell fate specification 10, 11, 12, cell polarity 13, 14 and cell proliferation 15, 16, 17. Considering this apparent complexity of cadherin function in morphogenesis, it is difficult to always clearly distinguish between the different morphogenetic functions of cadherins. In this review, we aim at dissecting the different major functions of cadherins in cell–cell contact formation and stabilization.

When cells contact each other, cadherins from the opposing cells located at the site of contact form *trans*-bonds across the contact. Once engaged in *trans*-bonds, cadherins can regulate the formation of the cell–cell contact in three distinct ways: by reducing the local interfacial tension directly through adhesion tension and indirectly through signaling to the actomyosin cytoskeleton, and by establishing the mechanical coupling of contacting cells.

### Adhesion Tension Function of Cadherins

At the macroscopic level, cell and tissue shapes are regulated by surface tension-like properties (Box 1) 18, 19, 20, 21, 22, 23. Similar to the rounding up of liquids, surface tension-like properties can explain the rounding up of cells during cytokinesis 24, 25 or of tissues when explanted 23, 26, 27. Furthermore, in analogy to the arrangement of soap bubbles, surface tension-like properties have been used to explain cell packing in epithelia 13, 28, 29, 30 and the configuration of a subset of cells within tissues 1, 31. Reducing the surface tension of a specific interface, such as the cell–cell contact, tends to increase its size. One function of cadherin in cell–cell adhesion is to promote contact formation by directly reducing the surface tension at the cell–cell interface via adhesion tension. The adhesion tension arises from cadherin binding over the contact thereby generating a negative tension that expands the contact area 32, 33.

Box 1 **Glossary.**

**Surface or interfacial tension**: the energy required to decrease by a unit area a given surface (or interface). Similar as to liquids, surface tension gives cells and tissues an apparent stiffness, which resists mechanical stresses. Tensions are measured in N.m^-1^. Typical tensions associated with cells range from 10^-5^ –10^-4^ N.m^-1^ 45, 46, 113 while tissue surface tensions are usually found in the 10^-3^ N.m^-1^ range 23, 114.

**Cortex tension**: the contribution of the actomyosin cortex to the tension of a given surface or interface. In isolated cells or at contact-free surfaces in a tissue, it generally constitutes most of the tension 33, 45, 46, 113.

**Adhesion tension**: the contribution of adhesion molecules to the tension of a given interface. Adhesion molecules are typically expected to promote the expansion of cell–cell contacts. Therefore, adhesion tension is expected to be a negative tension or, as formulated above for surface tension, it is the energy required to increase a given interface by a unit area.

**Adhesion coupling**: the force opposed by adhesion molecules to intra- or extra-cellular forces. It can be studied by measuring the force required to separate adhesion molecules (in the pN range 115, 116) or cells (in the nN range 33, 46, 95).

**Adhesion signaling**: the chemical modifications resulting from cadherin *trans*-binding. Signaling from cadherins can have consequences on a broad range of cellular processes, from rapid cytoskeletal rearrangements, which can modify interfacial tension 33, 34 and adhesion coupling 81, 82, to the long-term differentiation of tissues [11].

**Figure 1:**
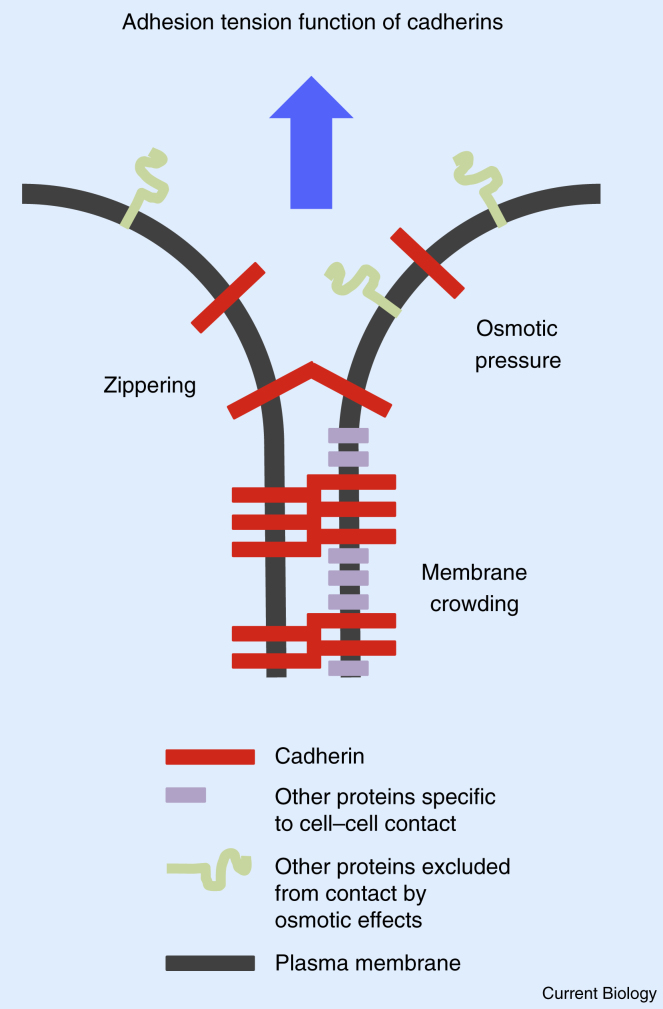
Adhesion tension. Upon cadherin *trans*-binding, opposing plasma membranes are brought closer together, thereby expanding the contact through zippering. Accumulation of cadherins and other proteins at the contact zone can also result in membrane crowding and expansion of the contact zone *via* lateral pressure. Finally, cadherin accumulation at the contact zone might result in an osmotic-driven movement of proteins outside of the contact zone, resulting in lateral pressure shrinking the contact. The combination of these effects results in the adhesion tension function of cadherins in cell–cell adhesion, which has a direct effect on the surface tension at the cell–cell contact and thereby regulates its size.

At the microscopic scale, adhesion tension is thought to originate from the chemical binding of cadherins, which is energetically favorable. As a consequence of binding, cadherins accumulate at the cell–cell contact where they are stabilized, because for cadherins to exit the contact zone, the cadherin *trans*-bond would need to be broken. Cadherin accumulation at the contact might influence contact size in several ways: cadherins could promote contact expansion via zippering of the contact edge (Figure 1). Consistent with this, accumulations of cadherins are often found at the edges of the cell–cell contact 33, 34. Zippering of the cell–cell contact by cadherins could be achieved by shortening the cadherin *trans*-bond length after a catch phase and/or stabilizing random encounters of opposing plasma membranes 35, 36. Alternatively or in addition, cadherins could control contact size via protein crowding (Figure 1). The crowding of proteins at the cell–cell contact can lead to the building of a lateral pressure in the plasma membrane [37], which forces the contacting plasma membrane to spread. Such lateral pressure from protein crowding is unlikely to arise solely from the action of cadherins, as cadherins commonly cluster and do not occupy the whole membrane surface of the cell–cell contact 33, 34, 38. However, other proteins associated with cadherins, or proteins that require cadherins to accumulate at the cell–cell contact could exert a lateral pressure and expand the contact zone. Finally, accumulation of cadherins that cannot exit the cell–cell contact could build an osmotic pressure that leads to the depletion of unbound cadherins and other proteins that are not yet sequestered at the cell–cell contact (Figure 1). Such osmotic depletion of unbound adhesion molecules would be expected to decrease the lateral pressure within the cell–cell contact and thus reduce adhesion tension and limit contact expansion.

While the exact microscopic mechanisms underlying the generation of macroscopic adhesion tension are yet unknown, several attempts were made to measure or calculate adhesion tension. For instance, contact separation experiments have been used to obtain information about the adhesion energy at cell–cell contacts. Adhesion energy is the work required to build or cleave an area of contact at the surface of passive materials and scales with adhesion tension, granted that the process of adhesion is reversible, *i.e.,* that the energy of formation and separation of an adhesive contact are the same. However, while the assumption that the energy of formation and separation of an adhesive contact are the same was validated for passive viscoelastic materials 39, 40, this might be different for living cells [33]. In fact, cell–cell adhesion is an active process that involves extensive molecular reorganization of the contact zone (see below), suggesting that the energies associated with contact formation and separation are different. It is thus questionable whether measuring the energy of cell–cell contact cleavages represents a suitable approach for obtaining information about the adhesion energy involved in contact formation.

More recently, the shape of adhering cells was used to directly calculate adhesion tension by determining the interfacial tension at the cell–cell contact, to which adhesion tension contributes. In *Drosophila* embryos, the analysis of cell shapes using sophisticated image analysis tools, together with modeling of cell mechanics, allowed determining the interfacial tension at cell–cell junctions 41, 42. In zebrafish embryos, the specific contribution of adhesion tension to the interfacial tension at the cell–cell contact was determined by measuring the interfacial tension at the contact in the presence and absence of adhesion tension [33]. Notably, adhesion tension was found to contribute only little to the total interfacial tension at the contact between these cells. This suggests that adhesion tension plays only a minor role in setting the cell–cell contact size. Future experiments addressing the contribution of adhesion tension in other cell types will be needed to determine whether adhesion tension can also play a more prominent function in cell–cell contact formation and stabilization.

### Signaling Function of Cadherins

Besides adhesion tension, cells can also use different strategies to reduce interfacial tension at the cell–cell contact and thereby increase the size of the contact. Actomyosin contractility is a decisive factor influencing surface tension of cells in a broad variety of organisms 43, 44, 45, 46. Once cells contact each other, they often reorganize their actomyosin cytoskeleton at the cell–cell interface [34], resulting in a reduction of interfacial tension at the contact, thereby expanding the contact size (Box 1) [33]. Contact-mediated reorganization of the actomyosin cytoskeleton is commonly attributed to signaling from the cadherin adhesion complex [47], although other cadherin-independent processes also might control the organization of the actomyosin cytoskeleton at the contact 48, 49.

**Figure 2:**
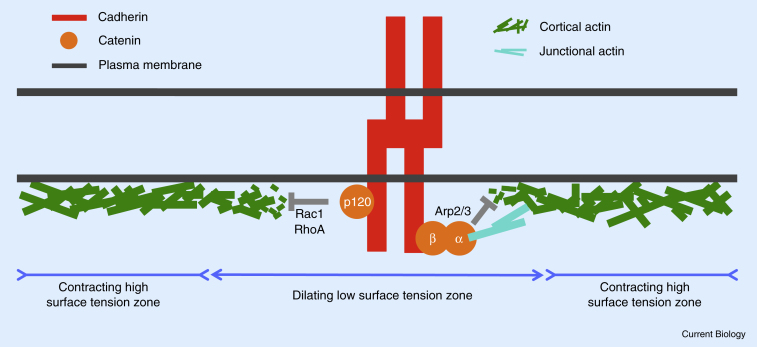
Adhesion signaling. Cadherin *trans*-binding triggers local signaling at the contact. This signaling is mediated by p120-catenin activating Rac1 and inhibiting RhoA, and by α-catenin interfering with the function of Arp2/3 in polymerizing actin. Cadherin-mediated signaling is thought to disrupt the contractile actomyosin cortex at the contact, thereby lowering cell–cell interfacial tension and expanding the contact.

Upon *trans*-binding of cadherins, the local chemistry changes at the cell–cell contact (Figure 2) [50]. For example, plasma membrane phosphatidylinositol (3,4,5)-trisphosphate (PIP_3_) accumulates, which in turn leads to local activation of Rac1 at the contact 34, 47. Likewise, p120-catenin binds to cadherins engaged in *trans*-binding, leading to local down-regulation of RhoA [51]. Also, β-catenin becomes recruited to the cadherin adhesion complex at the cell–cell contact and competes with Arp2/3 [52] and/or recruits RhoGEFs by interacting with centralspindlin [53]. Since both Arp2/3 and Rho GTPases, such as RhoA and Rac1, are known regulators of the actin cytoskeleton, changing their function leads to alterations in cytoskeleton organization at the cell–cell contact and, consequently, defects in contact formation 34, 51, 52.

Taken together, cadherins, in particular by recruiting catenins, trigger signals that modulate the actomyosin cytoskeleton at the cell–cell contact. Given that interfacial tension at the cell–cell contact is strongly influenced by actomyosin contractility, cadherin-mediated signaling is thus expected to modulate cell–cell contact interfacial tension and thus contact formation (Figure 2).

Cell–cell contact formation is typically followed by contact maturation. In epithelial cells, cell–cell contact maturation involves the establishment and stabilization of apico-basal cell polarity and the formation of tight junctions [54]. Interestingly, while the cortical actomyosin cytoskeleton is usually reduced at the contact during initial cell–cell contact formation and expansion [34], junctional myosin appears to be essential for epithelial contact maturation 55, 56. Myosins can have distinct functions in mature epithelial junctions with non-muscle myosin 2 heavy chains A and B controlling cadherin clustering and actin dynamics, respectively. These distinct functions of myosin isoforms in epithelial junctions are thought to be regulated by the GTPases RhoA and Rap1 [57]. Recently, Rap1 was also identified as a regulator of epithelial folding by regulating cytoskeletal anchoring of cadherins through β-catenin [8]. Further studies will be needed to understand the temporal and spatial regulation of the molecular composition of the adhesion complex and associated cytoskeleton during cell–cell contact maturation.

Cells not only make but also break contacts with their neighboring cells during various morphogenetic events, such as collective cell migration and cell ingression 4, 9, 58, 59, 60, 61. During neural crest cell migration in zebrafish and *Xenopus laevis*, cells separate from their neighbors through contact inhibition of locomotion by activating RhoA and inhibiting Rac1 at the cell–cell contact 62, 63. Also, during convergence and extension movements in *Drosophila*, *Xenopus* and zebrafish gastrulation, Rho GTPase-signaling and increased myosin contractility at the cell–cell contacts regulate neighbor exchanges 48, 59, 64, 65, 66. Notably, these are the opposite mechanisms of those taking place during cell–cell contact formation. Thus, contact separation may be mediated by the same signaling mechanisms controlling cell–cell interfacial tension as it is in contact formation. It is still unclear how cadherin-mediated signaling controls both contact growth and shrinkage, but different cadherins might display specific functions in these processes. For instance, N-cadherin is specifically required for mediating contact inhibition of locomotion in neural crest cells [63]. Other adhesion molecules, such as the atypical cadherins Fat and Dachs, or cadherin-associated proteins, such as nectins and DDR1, also seem to control cell–cell contact formation by regulating actomyosin contractility at the contact 13, 67, 68, 69. To understand how cadherin-mediated signaling differently controls contact growth and shrinkage, it will be key to identify and characterize contact- and cell type-specific effector proteins modulating the actomyosin cortex.

### Mechanical Scaffolding Function of Cadherins

By influencing cell–cell interfacial tension, the contractile actomyosin cytoskeleton plays a key role in contact formation and stabilization. In addition, the actomyosin cytoskeleton exerts forces pulling on the cell–cell junctions 9, 70, 71, 72. Recently, these pulling forces were directly visualized using a stretchable cadherin–FRET sensor [73]. Pulling forces generated by the actomyosin cytoskeleton are resisted by the mechanical coupling function of the cadherin adhesion complex at the contact, thereby preventing contact separation (Box 1) [2].

To mediate the mechanical coupling between cells (the force or load that adhesive bonds can withstand), cadherins anchor to the actomyosin cytoskeleton. When under mechanical load, cadherins can transmit through their cytoskeletal anchor forces to the whole interconnected cytoskeleton. The ‘basic’ cytoskeletal anchoring of cadherins is thought to be mediated by β- and α-catenin 10, 74, 75.

**Figure 3:**
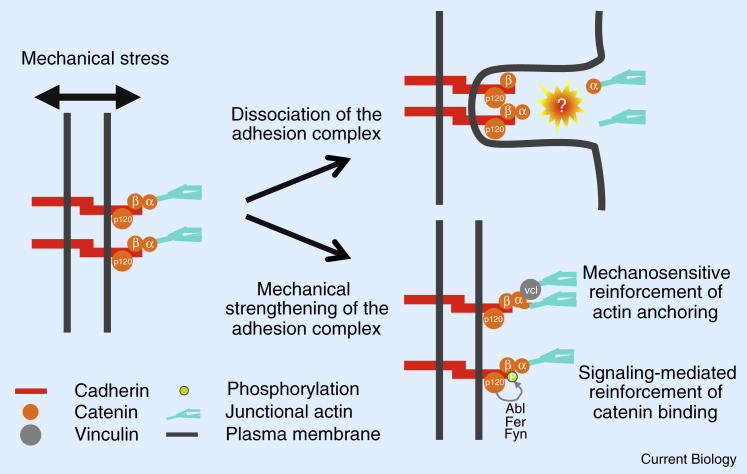
Adhesion coupling. When mechanically challenged, the adhesion complex dissociates at its cytoplasmic side. It is not clear whether dissociation occurs between α-catenin and β-catenin or downstream of them. To prevent dissociation, the intracellular part of the adhesion complex can be strengthened either *via* chemical signaling from p120-catenin, which stabilizes β-catenin–α-catenin interaction or *via* mechanosensing of α-catenin, which unfolds and recruits vinculin, which again strengthens anchoring to the actin cytoskeleton.

Interestingly, within the cadherin mechanical scaffold, the weakest link appears to be in the cytoplasmic rather than in the extracellular part of the scaffold (Figure 3). Upon separation, cells often remain connected by plasma membrane tethers at the base of which cadherins accumulate, suggesting that detachment occurs within the cell and that cadherins *trans*-bonds remain intact 33, 60, 76, 77. Similarly, retraction fibers at the back of migrating cells or attached to dividing cells and adhesion plaques suggest that cell-matrix attachment is limited by the anchoring of integrins to the actin cytoskeleton rather than by the integrin-matrix binding strength [78]. These observations suggest that morphogenetic processes that involve cell–cell separation, such as collective cell migration and cell ingression, may be primarily influenced by the coupling of cadherins to the actomyosin cytoskeleton rather than by the strength of cadherin *trans*-bonds. Therefore, the regulation of the cytoskeletal anchoring of cadherins is a critical process in morphogenesis.

Precisely which bond limits the cytoskeletal anchoring of cadherins remains unclear. Interestingly, the fusion of E-cadherin and α-catenin can efficiently substitute for a loss of α-catenin and rescue cell–cell adhesion and embryonic development in *Drosophila* α-catenin or E-cadherin mutants 10, 75. This suggests that E-cadherin, β- and α-catenin unbinding is not required for efficient separation of cell–cell contacts and that the cadherin scaffold can break downstream of α-catenin (Figure 3). However, exogenously forced cell–cell contact separation leads to the detachment of the adhesion complex from the actomyosin cytoskeleton between β- and α-catenin, suggesting that the weakest link is upstream of α-catenin [33].

The mechanical scaffolding function of cadherins can be regulated via chemical signaling. Different kinases (Abl, Fer, Fyn) were shown to regulate the interaction of β- and α-catenin via tyrosine phosphorylation of β-catenin 79, 80. p120-catenin also plays an important role in regulating the phosphorylation of β-catenin upstream of these kinases 81, 82. However, the p120-catenin binding site of E-cadherin appears to be dispensable for normal *Drosophila* oogenesis and embryonic development [83].

Cytoskeletal anchoring of cadherins can also be achieved by recruiting additional actin-binding proteins such as vinculin and/or EPLIN 84, 85. Interestingly, recruitment of these actin-binding proteins is mechanosensitive 71, 84, 86, 87. This mechanosensitive recruitment of additional actin binders enables cells to adapt the strength of their mechanical scaffolding function of cadherins to the magnitude of external and internal mechanical stresses (Figure 3). At the molecular level, mechanosensing of the adhesion complexes is thought to be achieved by the unwinding of protein domains upon stretching, which would reveal cryptic binding sites [88]. When tension is applied to adherens junctions, vinculin-binding sites of α-catenin become exposed upon protein stretching, promoting vinculin recruitment to the adhesion complex [86]. Vinculin then further connects the adhesion complex to the actin cytoskeleton, thereby strengthening the cadherin mechanical scaffold 84, 86, 89. Interestingly, vinculin, whose structure closely resembles the one of α-catenin, may also stretch under mechanical load and thereby recruit further proteins to the cell junction in a mechanosensitive manner [90]. In addition to α-catenin and vinculin, β-catenin and the extracellular domain of cadherin can unfold upon tension, as suggested by both *in vitro* and *in silico* studies 91, 92, 93. However, to understand the molecular mechanisms of mechanosensing and subsequent reinforcement of the mechanical coupling of the adhesion complex, further information on the response of other molecules of the adhesion complex to mechanical load will be needed.

To probe the mechanical coupling of the adhesion complex within cells, several methods can be employed. Micropipette- 33, 94, 95, 96 or atomic force microscopy-based assays 5, 46 can be used to separate cell doublets and measure the corresponding separation force. The separation force reflects, among other cell properties, the mechanical coupling of the adhesion complex [97]. Alternatively, for cells adhering to an extracellular matrix, flexible substrates can be used to measure indirectly the tugging force at the cell–cell contact from the imbalances of the traction forces applied on the substrate 71, 72. Finally, flexible peptides inserted within proteins can be used to measure relative tension changes at the molecular level using FRET 73, 98, 99. It is not yet clear how much the latter methods reveal about the mechanical coupling strength of adhesion; however, contrary to mechanical cell–cell separating methods, they provide information on endogenous forces at cell–cell contacts rather than their response to exogenous forces.

### Dissecting the Different Functions of Cadherins in Morphogenesis

Cell sorting is a morphogenetic event driven by successive breaking and making of cell–cell contacts. Therefore, all three functions of cadherins described above are potentially involved in cell sorting. The differential adhesion hypothesis gives a thermodynamic description of cell sorting. In view of the differential adhesion hypothesis, cells reorganize their cell–cell contacts according to the amount of adhesion molecules available, until an energetic equilibrium is reached [100]. However, in various cases, the number of adhesion molecules expressed in cells does not scale with their sorting behavior, contrary to the predictions by the differential adhesion hypothesis 33, 46, 101, 102, 103.

In zebrafish, distinct germ layer progenitor cells sort primarily according to their ability to regulate the actomyosin cytoskeleton at the cell–cell contact and the cytoskeletal anchoring strength of cadherins [33]. Notably, the contribution of adhesion tension to progenitor cell sorting appears to be negligible when compared to cytoskeletal reorganization regulating cell–cell contact formation. Therefore, cadherins appear to primarily function in zebrafish germ-layer progenitor-cell sorting through their signaling and mechanical coupling functions rather than *via* their adhesion tension function.

In *Xenopus*, cadherins do not seem to be the only molecules at the cell–cell contact regulating sorting of germ layer progenitors 60, 101. During gastrulation, ephrins were shown to regulate contact repulsion between ectoderm and mesoderm [60]. There, the regulation of contact repulsion is mediated by the small GTPases Rac and RhoA, which in turn might modulate the actomyosin cytoskeleton. Alternatively, ephrins were shown to regulate cell–cell adhesion via ADAM10-mediated cleavage of the extracellular domain of E-cadherin [104]. Similarly to the situation in *Drosophila* and zebrafish 2, 33, contact separation of germ layer progenitors in *Xenopus* leads to the formation of membrane tethers, suggesting that cadherin-mediated cell–cell adhesion breaks in the cytoplasmic part of the cadherin adhesion complex [60].

Another widespread morphogenetic event in which all three functions of cadherins are at play is epithelial morphogenesis. During epithelial morphogenesis, tissue integrity needs to be preserved. For this, mechanical coupling of the junctional adhesion complex is critical. In fact, when cytoskeletal coupling of the junctional adhesion complex is affected, such as in the ventral furrow cells of *Drosophila* β-catenin mutants, adhesion complexes are extruded from the cell body and morphogenesis halts [2]. Cytoskeletal anchoring of cadherins is also critical for maintaining tissue integrity during cytokinesis within *Drosophila* epithelial tissues. At early stages of cytokinesis, cadherins coupled to the cytoskeleton pull the cytokinetic actomyosin ring towards the apical junction of the dividing cells. Only shortly before a new cell–cell junction between the daughter cells is formed, cadherins disconnect from the actomyosin ring [105].

Besides mechanical coupling, cadherins also regulate interface tension at cell–cell boundaries to regulate cell shape and positioning. In the *Drosophila* retina, for example, cell shape is regulated by the presence of E-cadherin and/or N-cadherin [1], determining differences in interfacial tensions between these cells [31]. Similarly, in the *Drosophila* wing disc, adhesion tension and cortex tension at apical junctions were proposed to regulate epithelial packing 28, 106. However, whether cadherins directly lower interfacial tension via adhesion tension or indirectly by reorganizing the actomyosin cortex remains to be investigated. Similarly, in the extending germ band of the *Drosophila* embryo, cadherins accumulate at expanding cell–cell junctions, whereas they are depleted from shrinking boundaries 106, 107. At shrinking junctions, actomyosin appears to be the main factor determining interfacial tension controlling cell neighbor exchange 43, 59, 108. However, the specific contribution of cadherins to the interfacial tension in this process is still unknown. Recently, the differential localization of cadherins along junctions with different orientations was shown to direct the flow of actin and myosin from the cell apex towards those junctions. Interestingly, actomyosin flows towards the junctions containing less E-cadherin, suggesting that the flow results from unbalanced coupling of the actomyosin network to the junction rather than its total coupling strength [59]. It will be interesting to further investigate how cadherins, through their distinct functions, can direct flows of actomyosin in different morphogenetic movements 9, 109, 110.

### Conclusion

Since their discovery 111, 112, the functions of cadherins in morphogenesis have been intensively studied. Recent technological advances gave us a more comprehensive biophysical description of how cadherins function in morphogenesis. It appears that cadherins can have rather distinct functions in cell adhesion and that therefore using the term ‘adhesion’ in conjunction with cadherin function might be confusing. We thus would like to propose to use the term ‘adhesion tension’ when referring to the direct effect of cadherins on interfacial tension (Box 1). For the indirect effect of cadherins on interfacial tension through signaling to the cortical cytoskeleton, we would propose to use the term ‘adhesion signaling’. Finally, to refer to the function of cadherins in coupling the contractile cell cortices at the contact, the term ‘adhesion coupling’ might be most suitable. Obviously, any other terms might be equally good as long as they clearly distinguish between the specific adhesion functions of cadherins.

As a final note, we would like to emphasize that while cadherins clearly display different adhesion functions, it is experimentally very difficult to specifically regulate any of those functions alone. Changing adhesion signaling, for example, will predictably also alter adhesion coupling by affecting the cytoskeleton to which the adhesion complex is coupled. Future studies will have to address the interplay between the different functions of cadherins and their co-regulation in tissue morphogenesis.

## References

[1] Hayashi T., Carthew R. (2004). Surface mechanics mediate pattern formation in the developing retina. Nature.

[2] Martin A.C., Gelbart M., Fernandez-Gonzalez R., Kaschube M., Wieschaus E.F. (2010). Integration of contractile forces during tissue invagination. J. Cell Biol..

[3] Wang Y.-C., Khan Z., Kaschube M., Wieschaus E.F. (2012). Differential positioning of adherens junctions is associated with initiation of epithelial folding. Nature.

[4] Chihara D., Nance J. (2012). An E-cadherin-mediated hitchhiking mechanism for C. elegans germ cell internalization during gastrulation. Development.

[5] Arboleda-Estudillo Y., Krieg M., Stühmer J., Licata N.A., Muller D.J., Heisenberg C.-P. (2010). Movement directionality in collective migration of germ layer progenitors. Curr. Biol..

[6] Kardash E., Reichman-Fried M., Maître J.-L., Boldajipour B., Papusheva E., Messerschmidt E.-M., Heisenberg C.-P., Raz E. (2010). A role for Rho GTPases and cell–cell adhesion in single-cell motility in vivo. Nat. Cell Biol..

[7] Godt D., Tepass U. (1998). Drosophila oocyte localization is mediated by differential cadherin-based adhesion. Nature.

[8] Wang Y.-C., Khan Z., Wieschaus E.F. (2013). Distinct Rap1 activity states control the extent of epithelial invagination via α-catenin. Dev. Cell.

[9] Roh-Johnson M., Shemer G., Higgins C.D., McClellan J.H., Werts A.D., Tulu U.S., Gao L., Betzig E., Kiehart D.P., Goldstein B. (2012). Triggering a cell shape change by exploiting preexisting actomyosin contractions. Science.

[10] Sarpal R., Pellikka M., Patel R.R., Hui F.Y.W., Godt D., Tepass U. (2012). Mutational analysis supports a core role for Drosophila α-Catenin in adherens junction function. J. Cell Sci..

[11] Stephenson R.O., Yamanaka Y., Rossant J. (2010). Disorganized epithelial polarity and excess trophectoderm cell fate in preimplantation embryos lacking E-cadherin. Development.

[12] Lorthongpanich C., Doris T.P.Y., Limviphuvadh V., Knowles B.B., Solter D. (2012). Developmental fate and lineage commitment of singled mouse blastomeres. Development.

[13] Bosveld F., Bonnet I., Guirao B., Tlili S., Wang Z., Petitalot A., Marchand R., Bardet P.-L., Marcq P., Graner F. (2012). Mechanical control of morphogenesis by Fat/Dachsous/Four-jointed planar cell polarity pathway. Science.

[14] Wang Y., Kaiser M.S., Larson J.D., Nasevicius A., Clark K.J., Wadman S.A., Roberg-Perez S.E., Ekker S.C., Hackett P.B., McGrail M. (2010). Moesin1 and Ve-cadherin are required in endothelial cells during in vivo tubulogenesis. Development.

[15] Nelson C.M., Chen C.S. (2003). VE-cadherin simultaneously stimulates and inhibits cell proliferation by altering cytoskeletal structure and tension. J. Cell Sci..

[16] Kim N.-G., Koh E., Chen X., Gumbiner B.M. (2011). E-cadherin mediates contact inhibition of proliferation through Hippo signaling-pathway components. Proc. Natl. Acad. Sci. USA.

[17] Schlegelmilch K., Mohseni M., Kirak O., Pruszak J., Rodriguez J.R., Zhou D., Kreger B.T., Vasioukhin V., Avruch J., Brummelkamp T.R. (2011). Yap1 acts downstream of α-catenin to control epidermal proliferation. Cell.

[18] Manning M.L., Foty R.A., Steinberg M.S., Schoetz E.-M. (2010). Coaction of intercellular adhesion and cortical tension specifies tissue surface tension. Proc. Natl. Acad. Sci. USA.

[19] Manning M.L., Foty R.A., Steinberg M.S., Schoetz E.-M. (2011). Reply to Krens *et al*: Cell stretching may initiate cell differentiation. Proc. Natl. Acad. Sci. USA.

[20] Krens S.F.G., Möllmert S., Heisenberg C.-P. (2011). Enveloping cell-layer differentiation at the surface of zebrafish germ-layer tissue explants. Proc. Natl. Acad. Sci. USA.

[21] Brodland G. (2002). The Differential Interfacial Tension Hypothesis (DITH): a comprehensive theory for the self-rearrangement of embryonic cells and tissues. J. Biomech. Eng..

[22] Stirbat T.V., Mgharbel A., Bodennec S., Ferri K., Mertani H.C., Rieu J.-P., Delanoë-Ayari H. (2013). Fine tuning of tissues' viscosity and surface tension through contractility suggests a new role for α-catenin. PLoS ONE.

[23] Foty R.A., Pfleger C.M., Forgacs G., Steinberg M.S. (1996). Surface tensions of embryonic tissues predict their mutual envelopment behavior. Development.

[24] Kunda P., Pelling A., Liu T., Baum B. (2008). Moesin controls cortical rigidity, cell rounding, and spindle morphogenesis during mitosis. Curr. Biol..

[25] Stewart M.P., Helenius J., Toyoda Y., Ramanathan S.P., Muller D.J., Hyman A.A. (2011). Hydrostatic pressure and the actomyosin cortex drive mitotic cell rounding. Nature.

[26] Ninomiya H., Winklbauer R. (2008). Epithelial coating controls mesenchymal shape change through tissue-positioning effects and reduction of surface-minimizing tension. Nat. Cell Biol..

[27] Brodland G.W., Yang J., Sweny J. (2009). Cellular interfacial and surface tensions determined from aggregate compression tests using a finite element model. HFSP J..

[28] Farhadifar R., Röper J.-C., Aigouy B., Eaton S., Jülicher F. (2007). The influence of cell mechanics, cell–cell interactions, and proliferation on epithelial packing. Curr. Biol..

[29] Salbreux G., Barthel L.K., Raymond P.A., Lubensky D.K. (2012). Coupling mechanical deformations and planar cell polarity to create regular patterns in the zebrafish retina. PLoS Comput. Biol..

[30] Aigouy B., Farhadifar R., Staple D.B., Sagner A., Röper J.-C., Jülicher F., Eaton S. (2010). Cell flow reorients the axis of planar polarity in the wing epithelium of Drosophila. Cell.

[31] Kafer J., Hayashi T., Maree A., Carthew R., Graner F. (2007). Cell adhesion and cortex contractility determine cell patterning in the Drosophila retina. Proc. Natl. Acad. Sci. USA.

[32] Maître J.-L., Heisenberg C.-P. (2011). The role of adhesion energy in controlling cell–cell contacts. Curr. Opin. Cell Biol..

[33] Maître J.-L., Berthoumieux H., Krens S.F.G., Salbreux G., Jülicher F., Paluch E., Heisenberg C.-P. (2012). Adhesion functions in cell sorting by mechanically coupling the cortices of adhering cells. Science.

[34] Yamada S., Nelson W.J. (2007). Localized zones of Rho and Rac activities drive initiation and expansion of epithelial cell–cell adhesion. J. Cell Biol..

[35] Tollis S., Dart A.E., Tzircotis G., Endres R.G. (2010). The zipper mechanism in phagocytosis: energetic requirements and variability in phagocytic cup shape. BMC Syst. Biol..

[36] Weikl T.R., Asfaw M., Krobath H., Różycki B., Lipowsky R. (2009). Adhesion of membranes via receptor–ligand complexes: Domain formation, binding cooperativity, and active processes. Soft Matter.

[37] Stachowiak J.C., Schmid E.M., Ryan C.J., Ann H.S., Sasaki D.Y., Sherman M.B., Geissler P.L., Fletcher D.A., Hayden C.C. (2012). Membrane bending by protein-protein crowding. Nat. Cell Biol..

[38] Cavey M., Rauzi M., Lenne P., Lecuit T. (2008). A two-tiered mechanism for stabilization and immobilization of E-cadherin. Nature.

[39] Chu Y., Dufour S., Thiery J., Perez E., Pincet F. (2005). Johnson-Kendall-Roberts theory applied to living cells. Phys. Rev. Lett..

[40] Pierrat S., Brochard-Wyart F., Nassoy P. (2004). Enforced detachment of red blood cells adhering to surfaces: statics and dynamics. Biophys. J..

[41] Brodland G.W., Conte V., Cranston P.G., Veldhuis J., Narasimhan S., Hutson M.S., Jacinto A., Ulrich F., Baum B., Miodownik M. (2010). Video force microscopy reveals the mechanics of ventral furrow invagination in Drosophila. Proc. Natl. Acad. Sci. USA.

[42] Chiou K.K., Hufnagel L., Shraiman B.I. (2012). Mechanical stress inference for two dimensional cell arrays. PLoS Comput. Biol..

[43] Rauzi M., Verant P., Lecuit T., Lenne P. (2008). Nature and anisotropy of cortical forces orienting Drosophila tissue morphogenesis. Nat. Cell Biol..

[44] Mayer M., Depken M., Bois J.S., Jülicher F., Grill S.W. (2010). Anisotropies in cortical tension reveal the physical basis of polarizing cortical flows. Nature.

[45] Tinevez J.-Y., Schulze U., Salbreux G., Roensch J., Joanny J.-F., Paluch E. (2009). Role of cortical tension in bleb growth. Proc. Natl. Acad. Sci. USA.

[46] Krieg M., Arboleda-Estudillo Y., Puech P., Kafer J., Graner F., Muller D., Heisenberg C. (2008). Tensile forces govern germ-layer organization in zebrafish. Nat. Cell Biol..

[47] Perez T.D., Tamada M., Sheetz M.P., Nelson W.J. (2008). Immediate-early signaling induced by E-cadherin engagement and adhesion. J. Biol. Chem..

[48] Simões S., de M., Blankenship J.T., Weitz O., Farrell D.L., Tamada M., Fernandez-Gonzalez R., Zallen J.A. (2010). Rho-kinase directs Bazooka/Par-3 planar polarity during Drosophila axis elongation. Dev. Cell.

[49] Georgiou M., Marinari E., Burden J., Baum B. (2008). Cdc42, Par6, and aPKC regulate Arp2/3-mediated endocytosis to control local adherens junction stability. Curr. Biol..

[50] Zaidel-Bar R. (2013). Cadherin adhesome at a glance. J. Cell Sci..

[51] Anastasiadis P.Z., Moon S.Y., Thoreson M.A., Mariner D.J., Crawford H.C., Zheng Y., Reynolds A.B. (2000). Inhibition of RhoA by p120 catenin. Nat. Cell Biol..

[52] Benjamin J.M., Kwiatkowski A.V., Yang C., Korobova F., Pokutta S., Svitkina T., Weis W.I., Nelson W.J. (2010). AlphaE-catenin regulates actin dynamics independently of cadherin-mediated cell–cell adhesion. J. Cell Biol..

[53] Ratheesh A., Gomez G.A., Priya R., Verma S., Kovacs E.M., Jiang K., Brown N.H., Akhmanova A., Stehbens S.J., Yap A.S. (2012). Centralspindlin and α-catenin regulate Rho signalling at the epithelial zonula adherens. Nat. Cell Biol..

[54] Kishikawa M., Suzuki A., Ohno S. (2008). aPKC enables development of zonula adherens by antagonizing centripetal contraction of the circumferential actomyosin cables. J. Cell Sci..

[55] Miyake Y., Inoue N., Nishimura K., Kinoshita N., Hosoya H., Yonemura S. (2006). Actomyosin tension is required for correct recruitment of adherens junction components and zonula occludens formation. Exp. Cell Res..

[56] Shewan A., Maddugoda M., Kraemer A., Stehbens S., Verma S., Kovacs E., Yap A. (2005). Myosin 2 is a key Rho kinase target necessary for the local concentration of E-cadherin at cell–cell contacts. Mol. Biol. Cell.

[57] Smutny M., Cox H., Leerberg J., Kovacs E., Conti M., Ferguson C., Hamilton N., Parton R., Adelstein R., Yap A. (2010). Myosin II isoforms identify distinct functional modules that support integrity of the epithelial zonula adherens. Nat. Cell Biol..

[58] Montero J., Carvalho L., Wilsch-Brauninger M., Kilian B., Mustafa C., Heisenberg C. (2005). Shield formation at the onset of zebrafish gastrulation. Development.

[59] Rauzi M., Lenne P.-F., Lecuit T. (2010). Planar polarized actomyosin contractile flows control epithelial junction remodelling. Nature.

[60] Rohani N., Canty L., Luu O., Fagotto F., Winklbauer R. (2011). EphrinB/EphB signaling controls embryonic germ layer separation by contact-induced cell detachment. PLoS Biol..

[61] Speirs C.K., Jernigan K.K., Kim S.-H., Cha Y.I., Lin F., Sepich D.S., DuBois R.N., Lee E., Solnica-Krezel L. (2010). Prostaglandin Gbetagamma signaling stimulates gastrulation movements by limiting cell adhesion through Snai1a stabilization. Development.

[62] Carmona-Fontaine C., Matthews H., Kuriyama S., Moreno M., Dunn G., Parsons M., Stern C., Mayor R. (2008). Contact inhibition of locomotion in vivo controls neural crest directional migration. Nature.

[63] Theveneau E., Marchant L., Kuriyama S., Gull M., Moepps B., Parsons M., Mayor R. (2010). Collective chemotaxis requires contact-dependent cell polarity. Dev. Cell.

[64] Kwan K., Kirschner M. (2005). A microtubule-binding Rho-GEF controls cell morphology during convergent extension of Xenopus laevis. Development.

[65] Marlow F., Topczewski J., Sepich D., Solnica-Krezel L. (2002). Zebrafish Rho kinase 2 acts downstream of Wnt11 to mediate cell polarity and effective convergence and extension movements. Curr. Biol..

[66] Weiser D., Row R., Kimelman D. (2009). Rho-regulated Myosin phosphatase establishes the level of protrusive activity required for cell movements during zebrafish gastrulation. Development.

[67] Chang L.-H., Chen P., Lien M.-T., Ho Y.-H., Lin C.-M., Pan Y.-T., Wei S.-Y., Hsu J.-C. (2011). Differential adhesion and actomyosin cable collaborate to drive Echinoid-mediated cell sorting. Development.

[68] Hidalgo-Carcedo C., Hooper S., Chaudhry S.I., Williamson P., Harrington K., Leitinger B., Sahai E. (2011). Collective cell migration requires suppression of actomyosin at cell–cell contacts mediated by DDR1 and the cell polarity regulators Par3 and Par6. Nat. Cell Biol..

[69] Marcinkevicius E., Zallen J.A. (2013). Regulation of cytoskeletal organization and junctional remodeling by the atypical cadherin Fat. Development.

[70] de Rooij J., Kerstens A., Danuser G., Schwartz M., Waterman-Storer C. (2005). Integrin-dependent actomyosin contraction regulates epithelial cell scattering. J. Cell Biol..

[71] Liu Z., Tan J.L., Cohen D.M., Yang M.T., Sniadecki N.J., Ruiz S.A., Nelson C.M., Chen C.S. (2010). Mechanical tugging force regulates the size of cell–cell junctions. Proc. Natl. Acad. Sci. USA.

[72] Maruthamuthu V., Sabass B., Schwarz U.S., Gardel M.L. (2011). Cell-ECM traction force modulates endogenous tension at cell–cell contacts. Proc. Natl. Acad. Sci. USA.

[73] Borghi N., Sorokina M., Shcherbakova O.G., Weis W.I., Pruitt B.L., Nelson W.J., Dunn A.R. (2012). E-cadherin is under constitutive actomyosin-generated tension that is increased at cell–cell contacts upon externally applied stretch. Proc. Natl. Acad. Sci. USA.

[74] Desai R., Sarpal R., Ishiyama N., Pellikka M., Ikura M., Tepass U. (2013). Monomeric alpha-catenin links cadherin to the actin cytoskeleton. Nat. Cell Biol..

[75] Pacquelet A., Rørth P. (2005). Regulatory mechanisms required for DE-cadherin function in cell migration and other types of adhesion. J. Cell Biol..

[76] Tabdanov E., Borghi N., Brochard-Wyart F., Dufour S., Thiery J. (2009). Role of E-cadherin in membrane-cortex interaction probed by nanotube extrusion. Biophys. J..

[77] Gomperts M., Garcia-Castro M., Wylie C., Heasman J. (1994). Interactions between primordial germ cells play a role in their migration in mouse embryos. Development.

[78] Selhuber-Unkel C., Erdmann T., López-García M., Kessler H., Schwarz U.S., Spatz J.P. (2010). Cell adhesion strength is controlled by intermolecular spacing of adhesion receptors. Biophys. J..

[79] Tominaga J., Fukunaga Y., Abelardo E., Nagafuchi A. (2008). Defining the function of beta-catenin tyrosine phosphorylation in cadherin-mediated cell–cell adhesion. Genes Cells.

[80] Tamada M., Farrell D.L., Zallen J.A. (2012). Abl regulates planar polarized junctional dynamics through β-catenin tyrosine phosphorylation. Dev. Cell.

[81] Piedra J., Miravet S., Castaño J., Pálmer H.G., Heisterkamp N., García de Herreros A., Duñach M. (2003). p120 Catenin-associated Fer and Fyn tyrosine kinases regulate beta-catenin Tyr-142 phosphorylation and beta-catenin-alpha-catenin Interaction. Mol. Cell Biol..

[82] Thoreson M.A., Anastasiadis P.Z., Daniel J.M., Ireton R.C., Wheelock M.J., Johnson K.R., Hummingbird D.K., Reynolds A.B. (2000). Selective uncoupling of p120(ctn) from E-cadherin disrupts strong adhesion. J. Cell Biol..

[83] Pacquelet A., Lin L., Rorth P. (2003). Binding site for p120/delta-catenin is not required for Drosophila E-cadherin function in vivo. J. Cell Biol..

[84] le Duc Q., Shi Q., Blonk I., Sonnenberg A., Wang N., Leckband D., de Rooij J. (2010). Vinculin potentiates E-cadherin mechanosensing and is recruited to actin-anchored sites within adherens junctions in a myosin II-dependent manner. J. Cell Biol..

[85] Abe K., Takeichi M. (2008). EPLIN mediates linkage of the cadherin catenin complex to F-actin and stabilizes the circumferential actin belt. Proc. Natl. Acad. Sci. USA.

[86] Yonemura S., Wada Y., Watanabe T., Nagafuchi A., Shibata M. (2010). α-Catenin as a tension transducer that induces adherens junction development. Nat. Cell Biol..

[87] Taguchi K., Ishiuchi T., Takeichi M. (2011). Mechanosensitive EPLIN-dependent remodeling of adherens junctions regulates epithelial reshaping. J. Cell Biol..

[88] del Rio A., Perez-Jimenez R., Liu R., Roca-Cusachs P., Fernandez J.M., Sheetz M.P. (2009). Stretching single talin rod molecules activates vinculin binding. Science.

[89] Thomas W.A., Boscher C., Chu Y.-S., Cuvelier D., Martinez-Rico C., Seddiki R., Heysch J., Ladoux B., Thiery J.-P., Mège R.-M. (2013). α-Catenin and vinculin cooperate to promote high E-cadherin-based adhesion strength. J. Biol. Chem..

[90] Carisey A., Tsang R., Greiner A.M., Nijenhuis N., Heath N., Nazgiewicz A., Kemkemer R., Derby B., Spatz J., Ballestrem C. (2013). Vinculin regulates the recruitment and release of core focal adhesion proteins in a force-dependent manner. Curr. Biol..

[91] Valbuena A., Vera A.M., Oroz J., Menéndez M., Carrión-Vázquez M. (2012). Mechanical properties of β-catenin revealed by single-molecule experiments. Biophys. J..

[92] Liu R., Wu F., Thiery J.-P. (2012). Remarkable disparity in mechanical response among the extracellular domains of type I and II cadherins. J. Biomol. Struct. Dyn..

[93] Oroz J., Valbuena A., Vera A.M., Mendieta J., Gómez-Puertas P., Carrión-Vázquez M. (2011). Nanomechanics of the cadherin ectodomain “canalization” by Ca2+ binding results in a new mechanical element. J. Biol. Chem..

[94] Stockinger P., Maître J.-L., Heisenberg C.-P. (2011). Defective neuroepithelial cell cohesion affects tangential branchiomotor neuron migration in the zebrafish neural tube. Development.

[95] Chu Y., Thomas W., Eder O., Pincet F., Perez E., Thiery J., Dufour S. (2004). Force measurements in E-cadherin-mediated cell doublets reveal rapid adhesion strengthened by actin cytoskeleton remodeling through Rac and Cdc42. J. Cell Biol..

[96] Jegou A., Pincet F., Perez E., Wolf J., Ziyyat A., Gourier C. (2008). Mapping mouse gamete interaction forces reveal several oocyte membrane regions with different mechanical and adhesive properties. Langmuir.

[97] Seifert U. (2000). Rupture of multiple parallel molecular bonds under dynamic loading. Phys. Rev. Lett..

[98] Grashoff C., Hoffman B.D., Brenner M.D., Zhou R., Parsons M., Yang M.T., McLean M.A., Sligar S.G., Chen C.S., Ha T. (2010). Measuring mechanical tension across vinculin reveals regulation of focal adhesion dynamics. Nature.

[99] Conway D.E., Breckenridge M.T., Hinde E., Gratton E., Chen C.S., Schwartz M.A. (2013). Fluid shear stress on endothelial cells modulates mechanical tension across VE-cadherin and PECAM-1. Curr. Biol..

[100] Foty R., Steinberg M. (2005). The differential adhesion hypothesis: a direct evaluation. Dev. Biol..

[101] Ninomiya H., David R., Damm E.W., Fagotto F., Niessen C.M., Winklbauer R. (2012). Cadherin-dependent differential cell adhesion in Xenopus causes cell sorting in vitro but not in the embryo. J. Cell Sci..

[102] Shi Q., Chien Y., Leckband D. (2008). Biophysical properties of cadherin bonds do not predict cell sorting. J. Biol. Chem..

[103] Moore R., Cai K.Q., Escudero D.O., Xu X.-X. (2009). Cell adhesive affinity does not dictate primitive endoderm segregation and positioning during murine embryoid body formation. Genesis.

[104] Solanas G., Cortina C., Sevillano M., Batlle E. (2011). Cleavage of E-cadherin by ADAM10 mediates epithelial cell sorting downstream of EphB signalling. Nat. Cell Biol..

[105] Guillot C., Lecuit T. (2013). Adhesion disengagement uncouples intrinsic and extrinsic forces to drive cytokinesis in epithelial tissues. Dev. Cell.

[106] Levayer R., Pelissier-Monier A., Lecuit T. (2011). Spatial regulation of Dia and Myosin-II by RhoGEF2 controls initiation of E-cadherin endocytosis during epithelial morphogenesis. Nat. Cell Biol..

[107] Blankenship J., Backovic S., Sanny J., Weitz O., Zallen J. (2006). Multicellular rosette formation links planar cell polarity to tissue morphogenesis. Dev. Cell.

[108] Bertet C., Sulak L., Lecuit T. (2004). Myosin-dependent junction remodelling controls planar cell intercalation and axis elongation. Nature.

[109] Behrndt M., Salbreux G., Campinho P., Hauschild R., Oswald F., Roensch J., Grill S.W., Heisenberg C.-P. (2012). Forces driving epithelial spreading in zebrafish gastrulation. Science.

[110] Martin A., Kaschube M., Wieschaus E. (2009). Pulsed contractions of an actin-myosin network drive apical constriction. Nature.

[111] Hyafil F., Babinet C., Jacob F. (1981). Cell–cell interactions in early embryogenesis: a molecular approach to the role of calcium. Cell.

[112] Urushihara H., Takeichi M. (1980). Cell–cell adhesion molecule: identification of a glycoprotein relevant to the Ca2+-independent aggregation of Chinese hamster fibroblasts. Cell.

[113] Dai J., Ting-Beall H., Hochmuth R., Sheetz M., Titus M. (1999). Myosin I contributes to the generation of resting cortical tension. Biophys. J..

[114] Schötz E.-M., Burdine R.D., Jülicher F., Steinberg M.S., Heisenberg C.-P., Foty R.A. (2008). Quantitative differences in tissue surface tension influence zebrafish germ layer positioning. HFSP J..

[115] Perret E., Leung A., Feracci H., Evans E. (2004). Trans-bonded pairs of E-cadherin exhibit a remarkable hierarchy of mechanical strengths. Proc. Natl. Acad. Sci. USA.

[116] Baumgartner W., Hinterdorfer P., Ness W., Raab A., Vestweber D., Schindler H., Drenckhahn D. (2000). Cadherin interaction probed by atomic force microscopy. Proc. Natl. Acad. Sci. USA.

